# A potion for prolonged life? Germes’ recipe, a secret handwritten note from an 18th-century Swedish physician

**DOI:** 10.1186/s13002-025-00813-0

**Published:** 2025-10-22

**Authors:** Fabien Schultz, Tobias Niedenthal, Isabel Nicolai-Lorenz, Kandace Baez, Tabitha Iker, Leif-Alexander Garbe

**Affiliations:** 1 Ethnopharmacology and Zoopharmacognosy, Bernhard Nocht Institute for Tropical Medicine, Hamburg, Germany; 2 Department of Agriculture and Food Sciences, Neubrandenburg University of Applied Sciences, Neubrandenburg, Germany; 3 Forschergruppe Klostermedizin, Würzburg, Germany; 4 Freelancer Translator of Historical Texts, Wülfrath, Germany; 5 ZELT - Neubrandenburg Center for Nutrition and Food Technology gGmbH, Seestraße 7A, 17033 Neubrandenburg, Germany; 6 Department of Social Sciences, University of Hamburg, Allende-Platz 1, 20146 Hamburg, Germany

## Abstract

**Background:**

Toward the end of the late Middle Ages and into the early modern era, a variety of elixirs and potions for longevity with claimed medical effects were advertised by pharmacists and distributed throughout Europe. At the same time, there was an increasing emergence of handwritten commonplace books of home remedies, first among the aristocrats and rich merchants, then later among other populations, providing basic recipes for all types of common medical disorders. From a historical–pharmacological perspective, this study seeks to analyze a handwritten note encompassing a recipe detailing an herbal elixir for prolonged life that was written by a Swedish physician named Germes. It was discovered by the authors in an old herbal book purchased at a flea market in Germany. The note, composed of three pages, appears to be written in a type of German cursive handwriting. One study objective was to transcribe this note into Latin alphabet-based German and then further translate the document into modern English. Furthermore, this study aimed (a) to estimate the period of the note’s creation; (b) to contextualize Germes’ recipe in history regarding the contemporary understanding of the nature and effectiveness of such formulations; (c) to assess its uniqueness, and (d) to examine the criteria used by the original practitioner, and later by consumers, in order to evaluate the efficacy of such home remedies, while also analyzing how these perceptions evolved before and after creation of the handwritten primary source..

**Methods:**

The transcription process was performed through manual reading and inputting into a word processing software, with guidance from several manuals. Historical placement of the note was achieved through the assessment of the calligraphic handwriting and analysis of terms and words that were specific for a certain time period. Contextualization was performed through a review of various primary sources on plant medicine and contemporary pharmacy.

**Results:**

The analysis of the transcribed note made it possible to narrow down its creation to 1770–1820. It tells the story of Germes, whose secret elixir recipe was found in his jacket after he fatally fell off his horse at the age of 104, with his family members also living exceptionally long, which all accredits to the panacea described. The note contains accurate descriptions of its preparation and methods of administration, and Germes’ elixir can be used in the treatment of a variety of medical disorders. Several similar recipes for elixirs for longevity were identified in the literature, with a contemporary remedy called “Swedish Bitters” being nearly identical in terms of ingredient composition. The origins of “Swedish Bitters” were traced back to the late seventeenth century or early eighteenth century, where it rapidly gained popularity in the European market, particularly in Germany. This may accredit the handwritten note’s creation, modification, and embellishment through retelling.

## Introduction

Throughout human history, plants have been of great importance as sources of food and natural remedies. It is assumed that the use of medicinal plants goes back more than 60,000 years [1, 2]. Writings from the time of Emperor Shen Nung of China from around 2730–3000 BCE describe the medicinal use of plants such as ginseng, camphor, hemp, and aconite [3, 4]. The "Papyrus Ebers" were written around 1500 BCE and contain 877 recipes for the use of medicinal plants such as garlic, thyme, coriander, and aloe vera, including descriptions of the medicinal use of plant extracts like the poppy of opium and the oil of castor beans [3, 5]. Originally, the knowledge about the use of medicinal plants was passed on orally from one generation to another. However, old herbal books that contain information on plant use and its application in the treatment of medical disorders can now be considered vital sources for analyzing these ancient remedies under the lens of modern science.

Herbal books containing the valuable knowledge of ancient scholars, like Greek physicians Hippocrates (459–370 BCE) or Dioscorides during the first century, were translated several times over the course of time [4]. In the Middle Ages until about the twelfth century, the ancient knowledge of herbal remedies was documented by physicians, traditional healers, or nuns and monks in monasteries in the form of these herbal books. Important writings of the abbess, Hildegard von Bingen (1098–1179 CE), who studied the properties and effects of herbs, were also written during that time. With the invention of printing, knowledge could be widely disseminated and herbal books lost their status as luxury good for the upper classes by making the knowledge also accessible to the lower classes [6]. In traditional medicine, old remedies are still used today for a wide range of physical and psychological ailments, such as willow bark for analgesic, antipyretic, and anti-inflammatory effects, thyme for antibacterial and antiviral effects, or elderflower for diaphoretic and relaxing effects [7–9]. Although thousands of medicinal plants have been known since antiquity, most of them are no longer used in the Western world, as they seem to have disappeared or have been forgotten, and many have been entirely replaced by modern Western medicine [10–12].

This study covers the transcription, the translation, and the analysis of a handwritten note discovered in an old herbal book at a flea market in Eastern Germany. This primary source describes the handwritten recipe of an elixir from a Swedish physician by the name of Germes. The recipe was analyzed from a historical–ethnopharmacological perspective, and a historical context in German ethnomedicine was applied to the elixir and its ingredients utilizing other primary and secondary sources.

There are currently two methods for transcribing ancient documents from handwritten language styles to modern Latin alphabet-based German writing: (1) Handwriting Text Recognition (HRT) using modern artificial intelligence (AI) technologies, e.g., *Transkribus* software; and (2) a conventional approach using manual translation techniques, which requires expert-level knowledge and experience in historical handwriting [13, 14]. Since AI in this specific application is not yet at the stage where it can be considered reliable and error-free, using this conventional approach is mandatory especially considering it is not possible to check the accuracy of the AI results obtained but only to determine whether the text appears plausible [15, 16]. This does not meet the standards for scientific research. Therefore, this study seeks to transcribe the handwritten recipe by employing the conventional manual approach for transcriptions of old handwritten texts.

The objectives of this study are (1) to transcribe the primary source from handwritten German cursive into standard German using the Latin alphabet, then translating the text into modern English; (2) to determine the historical period of which the note was written; (3) to investigate whether the recipe represents a unique historical artifact or whether similar versions have been documented before or after its creation; and (4) to examine the criteria used by the original practitioner, and later by consumers, in order to evaluate the efficacy of the home remedy, while also analyzing how these perceptions evolved before and after creation of the handwritten primary source.

## Materials and methods

### Primary sources

**Fig. 1:**
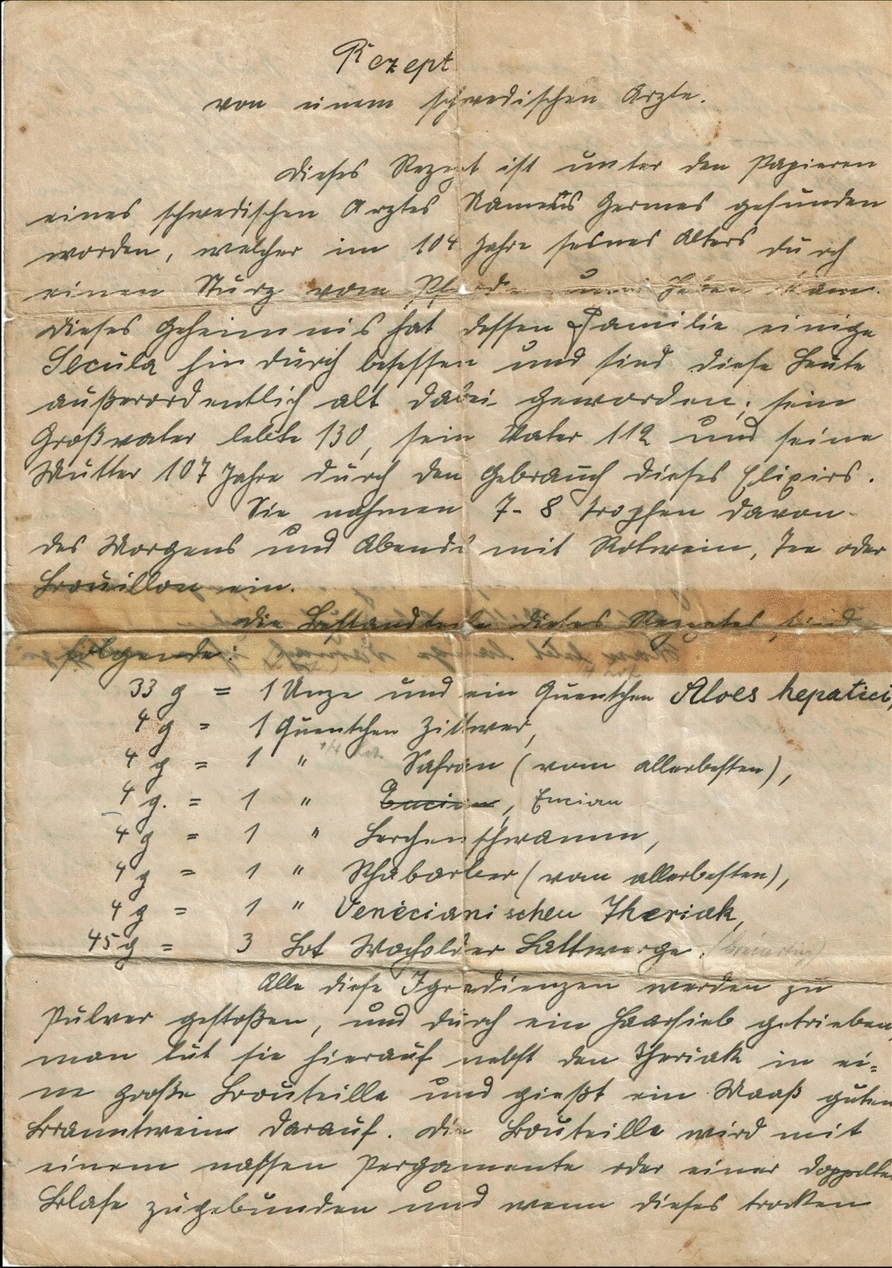
Page 1 of Germes’ recipe, a secret handwritten note from an 18th-century Swedish physician

**Fig. 2:**
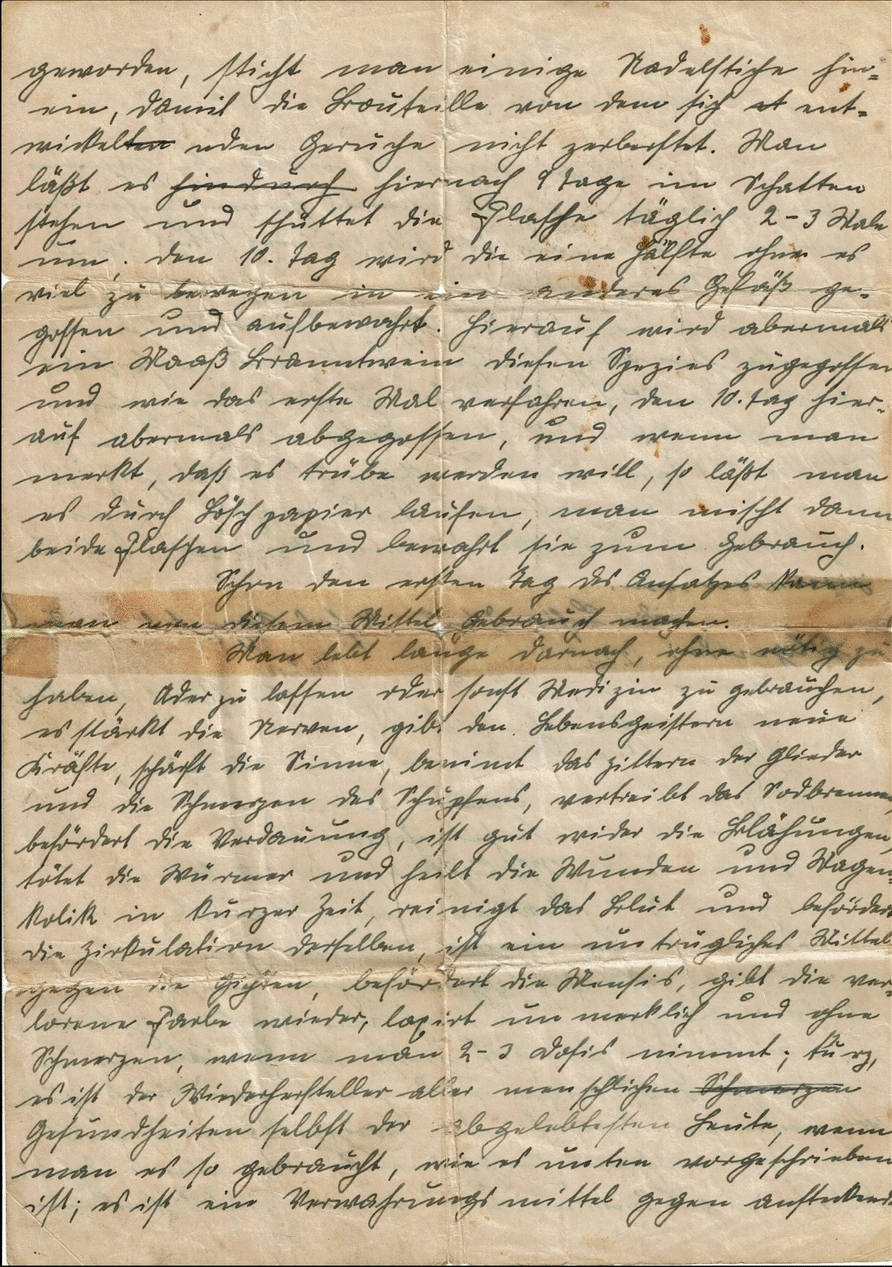
Page 2 of Germes’ recipe, a secret handwritten note from an 18th-century Swedish physician

**Fig. 3:**
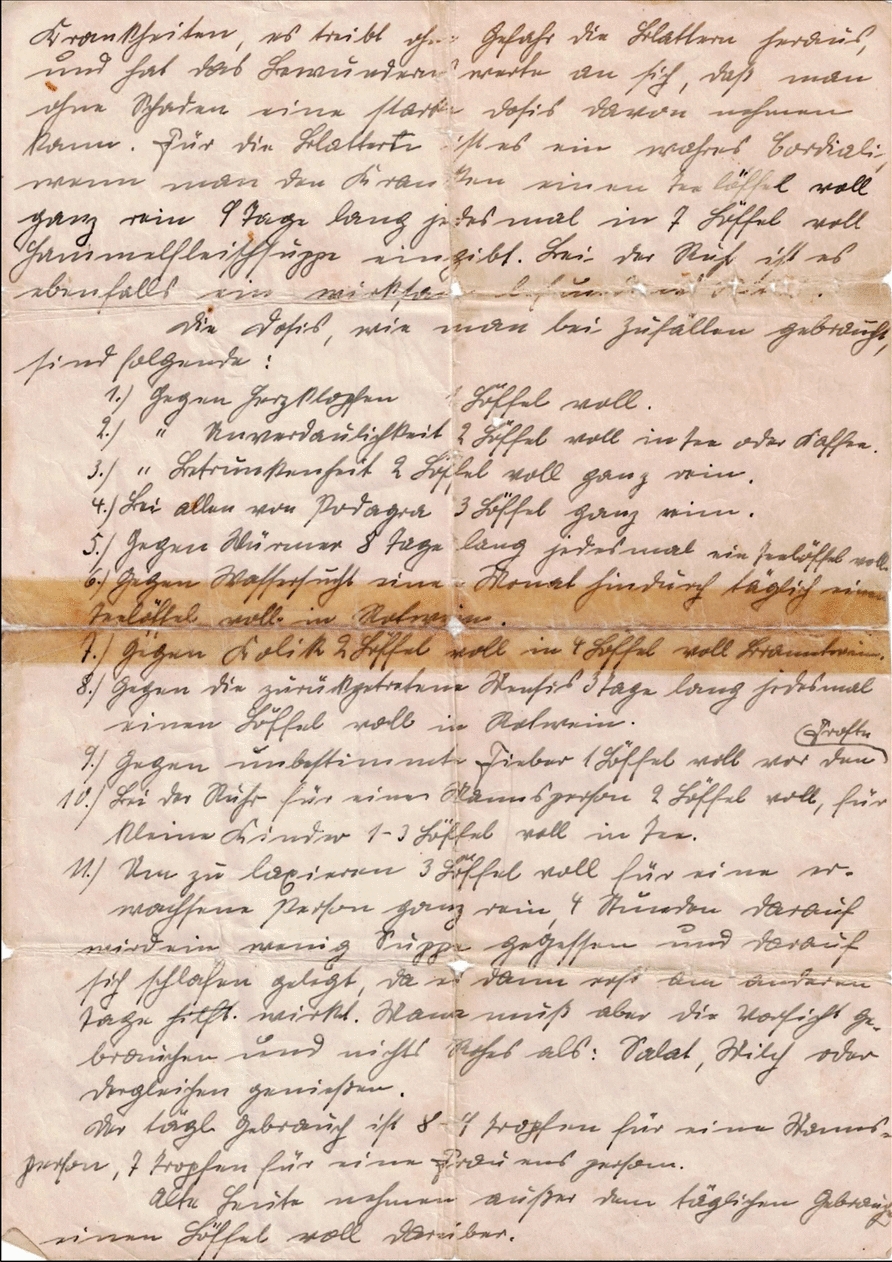
Page 3 of Germes’ recipe, a secret handwritten note from an 18th-century Swedish physician

The subject of study is a handwritten note, meticulously composed across three yellowed pages, shown in Figs. 1, 2, and 3. By chance, the authors acquired it at a flea market in Eastern Germany after discovering it tucked away in the pages of an old herbal book titled, *Das große illustrierte Kräuter-Buch*, written and published by Ferdinand Müller in 1860.

To contextualize the replication of recipe ingredients, this study incorporated various primary sources that shed light on the contemporary understanding of the nature, effectiveness, and mechanisms behind these preventive potions, as well as their implications for health, disease, mortality, and longevity. These include the following preserved original herbal books:

1. *Dispensatorium pharmaceuticum Austriaco-Viennense* [17].
2. * Das große illustrierte Kräuter-Buch* [18].
3. * Gemeinnütziges Kräuterbuch* [19].
4. *Sammlung vorzüglicher Hausmittel—gegen die meisten Krankheiten des Menschen sowie Beschreibung der heilkräftigsten Pflanzen* [20].
5. *Kräuterbuch—unsere Heilpflanzen in Wort und Bild* [21].
6. * Das Buch der Kräuter* [22].
7. *Lassels Kräutergold—mit Zusammenhänge über die Rückgewinnung der Blühenden Gesundheit durch naturgemäße Lebensführung* [23].

Moreover, the study examined the following herbal books, which were available in facsimile:

1. *Kreutterbuch* (Fuchs), 1543, reprint [24]
2. *Paradeißgärtlein*, 1588, reprint [25]
3. *Kreutterbuch* (Dioscorides), 1610, reprint [26]
4. *Kreutterbuch* (Matthioli), 1626, reprint [27]
5. *Kreuterbuch* (Tabernaemontanus), 1731, reprint [28]

In English, *Kreutterbuch*, *Kreuterbuch, Kräuter-Buch,* and *Kräuterbuch* can be translated as “herbal” or "herbal book."

The following primary sources were reviewed in this study, but were only available in digitized form:

1. *Lorscher Arzneibuch* [29]
2. *Dispensatorium Pharmacopolarum des Valerius Cordus* [30]
3. *Heidelberger Artzney-Buch* [31]
4. *D. Johann Schröders trefflich-versehene Medicin-Chymische Apotheke, Oder: Höchstkostbarer Arzeney-Schatz: Darinnen so wol einfache, als aus vielen Stücken bestehende, bewährteste Hilfmittel, welche nicht allein die dem Menschen zu kräftigster Gesundheit dienende Mineralien oder Bergsäffte, Pflantzen und Kräuter, sondern auch unterschiedliche Theile der Thiere betreffen, auf Medicinisch-Chymische Art kernreich erörtert werden, Dabey ferner zu mehrerm Verständnis aller Materien, ein zumahl höchstdienlich-nöthig-und nützlicher Schlüssel in Herrn D. Friedrich Hoffmanns Herrlichen Anmerkungen bestehend, befindlich, Deme noch über das um mehrer Vollkommenheit willen eine nehmhafte Anzahl Baconianisch-Cartesian-und Helmontianischer Vernufts-Gründen, wie auch rarester Arzneymittel der fürtreflichsten Herrn Medicorum, insonderheit des in gantz Sachsenland und anderer Orten höchstberühmten Herrn D. Iohann Michaelis beygefüget, und dann endlich das gantze preyßwürdige Werck mit einem pharmacevtischen Schatz der ruhmwürdigsten Arzneymittel dieser Zeit ausgeschmücket, in die Hochteutsche Sprache übersetzet und ans Licht gegeben wird* [32]
5. *L. Christoph. Hellwigs. Pract. Erff. auserlesenes Teutsch-Medicinisches Recept-Buch: Worinnen die heilsamsten und approbirtesten Artzeney-Mittel vor die meisten Kranckheiten der Mannes-Personen, Welche so wohl Ledige als Verehligte, absonderlich aber Gelehrte, Künstler und Handwercker, welche viel sitzen müssen, betreffen, aus berühmter Medicorum Schrifften… zusammen getragen… mit nöthigen Registern zum Druck befördert* [33]
6. *Johann Schröders Vollständige und nutzreiche Apotheke, Oder Trefflich-versehener Medicin-Chymischer höchst-kostbarer Artzney-Schatz: Nebst D. Friedrich Hoffmanns darüber verfasseten herrlichen Anmerkungen In fünff Bücher eingeheilt; Wider alle den menschlichen Leib anfeindende Kranckheiten aus derer ietziger Zeit berühmtesten Medicorum und gelehrtesten Männer… Schrifften… zusammen getragen und vermehret* [34]

### Transcription

The document, depicted in Figs. 1, 2 and 3, was transcribed into Latin alphabet-based German through manual reading and inputting into a word processing software, specifically Microsoft Word. Analysis and identification of German current script letters was performed with guidance from several books and manuals, including *Offinger* (1831), *Erlinghagen* (2011), *Braun* (2015), *Nöhrenberg* (2022), and *Kintzel* (2021) [35–39]. Original spellings and grammar have been obtained and used in the manuscript, even though certain terms are now considered wrong and/or are no longer in use in modern German language due to linguistic changes over the centuries. These contemporary terms have been discussed and analyzed in the results section (Section 3.2.3). For the units used in the handwritten note, it was only necessary to check their authenticity. This was done primarily through a literature review and one telephone consultation with the Austrian pharmacist and collector of historical pharmacy knowledge, Mag. pharm. Gilbert Zinsler (Pharmacy: Landschafts-Apotheke "Zum schwarzen Adler," 3580 Horn, Hauptplatz 14, Austria; E-mail: gilbert.zinsler@landschafts-apotheke-horn.at).

### Translation of the transcribed document

Regarding the translation of the German language transcription into modern English, the text has undergone some minor changes in order to align with today's language style, but its overall meaning has not been altered. This encompassed the employment and replacement of words and terms that are no longer in use or are unfamiliar to modern society. Old terms for different medical conditions from medieval and early modern German language were first identified and translated into modern German and then into their final contemporary English form using assistance of Höfler (1899) [40].

### Historical placement

Various aspects were taken into consideration in order to estimate the era of the recipe's creation. These were (a) the calligraphic style (“Deutsche Kanzleischrift” vs. “Kurrent” vs. “Sütterlin”) as well as the respective gradations of calligraphy development over the centuries; (b) the calligraphic style to investigate whether it was a skilled or unskilled scribe; (c) the spelling of specific words and the use of terms specific to a certain time period; (d) an investigation on whether it was a skilled or unskilled scribe based on the calligraphic style; and (e) the terms used both in the specialized fields of pharmacology and in civil language of certain time periods.

### Contextualization

The process of contextualization and its evaluation was achieved through reviewing old books on plant medicine and contemporary pharmacy. This process included, for example, whether the ingredients or a similar combination of ingredients of Germes’ recipe is replicated in other sources and how such a recipe would align within the understandings of the nature, efficacy, and action at the time of its creation. This approach also focused on the roles of contemporary magic, folklore, chemistry/alchemy, and sense of mystique of lineage in shaping historical beliefs about health, illness, mortality, and longevity.

### Ingredient classification

Ingredients mentioned in the recipe were reviewed, and today’s accepted scientific names for each plant species have been verified using the Kew Medicinal Plant Names Service (https://mpns.science.kew.org). Plant family assignments were conducted in accordance with The Angiosperm Phylogeny Group IV guidance [41].

## Results and discussion

### Transcription and translation of the primary source

The handwritten recipe was successfully translated into Latin alphabet-based German (see 3.1.1). To the utmost extent of the author's capacity and understanding, Section 3.1.2 showcases the successful translation of the German transcription of the recipe into modern English.

#### Latin alphabet-based German version

Page 1

**Rezept von einem schwedischen Arzte**

Dieses Rezept ist unter den Papieren eines schwedischen Arztes namens Germes gefunden worden, welcher im 104. Jahr seines Alters durch einen Sturz vom Pferde ums Leben kam. Dieses Geheimnis hat dessen Familie einige Secula hin durch besessen und sind diese Leute außerordentlich alt dabei geworden; sein Großvater lebte 130, sein Vater 112 und seine Mutter 107 Jahre durch den Gebrauch dieses Elixiers.

Sie nahmen 7–8 Tropfen davon des Morgens und Abends mit Rotwein, Tee oder Bouillon ein.

Die Bestandteile dieses Rezeptes sind folgende:

33 g = 1 Unze und ein Quentchen Aloes hepatici

4 g = 1 Quentchen Zittwer

4 g = 1 Quentchen Safran (vom allerbesten)

4 g = 1 Quentchen Encian

4 g = 1 Quentchen Lerchenschwamm

4 g = 1 Quentchen Rhabarber (vom allerbesten)

4 g = 1 Quentchen Venecianischen Theriak

45 g = 3 Lot Wacholder Lattwerge (breiartig)

Alle diese Ingredienzen werden zu Pulver gestoßen, und durch ein Haarsieb getrieben, man tut sie hierauf nebst dem Theriak in eine große Bouteille und gießt ein Maaß guten Branntwein darauf. Die Bouteille wird mit einem nassen Pergamente oder einer doppelten Blase zugebunden und wenn dieses trocken

Page 2

Geworden, sticht man einige Nadelstiche hinein, damit die Bouteille von dem sich entwickelnden Geruche nicht zerberstet. Man läßt es hiernach 9 Tage im Schatten stehen und schüttet die Flasche täglich 2–3 Male um, den 10. Tag wird die eine Hälfte, ohne es viel zu bewegen, in ein anderes Gefäß gegossen und aufbewahrt. Hierauf wird abermals ein Maaß Branntwein diesen Spezies zugegossen und wie das erste Mal verfahren, den 10. Tag hierauf abermals abgegossen, und wenn man merkt, daß es trübe werden will, so läßt man es durch Löschpapier laufen, man mischt dann beide Flaschen und bewahrt sie zum Gebrauch.

Schon den ersten Tag des Ansatzes kann man von diesem Mittel Gebrauch machen.

Man lebt lange darnach, ohne nötig zu haben, Ader zu lassen oder sonst Medizin zu gebrauchen, es stärkt die Nerven, gibt den Lebensgeistern neue Kräfte, schärft die Sinne, benimmt das Zittern der Glieder und die Schmerzen des Schupfens, vertreibt das Sodbrennen, befördert die Verdauung, ist gut wider die Blähungen, tötet die Würmer und heilt die Wunden und Magenkolik in kurzer Zeit, reinigt das Blut und befördert die Zirkulation desselben, ist ein untrügliches Mittel gegen die Gichten, befördert die Mensis, gibt die verlorene Farbe wieder, laxiert unmerklich und ohne Schmerzen, wenn man 2–3 Dosis nimmt; kurz, es ist der Wiederhersteller aller menschlichen Gesundheiten, selbst der abgelebtesten Leute, wenn man es so gebraucht, wie es wie es unten vorgeschrieben ist, es ist ein Verwahrungsmittel gegen ansteckende

Page 3

Krankheiten, es treibt ohne Gefahr die Blattern heraus, und hat das bewundernswerte an sich, daß man ohne Schaden eine starke Dosis davon nehmen kann. Für die Blattern ist es ein wahres Cordiali, wenn man den Kranken einen Teelöffel voll ganz rein 9 Tage lang jedesmal in 7 Löffel voll Hammelfleischsuppe eingibt. Bei der Ruhr ist es ebenfalls ein wirksam befundnes Mittel.

Die Dosis, wie man bei Zufällen gebraucht, sind folgende:

1. Gegen Herzklopfen 1 Löffel voll
2. Gegen Unverdaulichkeit 2 Löffel voll in Tee oder Kaffee
3. Gegen Betrunkenheit 2 Löffel voll ganz rein
4. Bei allen von Podagra 3 Löffel ganz rein
5. Gegen Würmer 8 Tage lang jedesmal ein Teelöffel voll
6. Gegen Wassersucht einen Monat hindurch täglich einen Teelöffel voll in Rotwein
7. Gegen Kolik 2 Löffel voll in 4 Löffel voll Branntwein
8. Gegen die zurückgetretene Mensis 3 Tage lang jedesmal einen Löffel voll in Rotwein
9. Gegen unbestimmte Fieber 1 Löffel voll vor den Froste
10. Bei der Ruhr für einen Mannsgroßen 2 Löffel voll, für kleine Kinder 1–3 Löffel voll in Tee
11. Um zu laxieren 3 Löffel voll für eine erwachsene Person ganz rein, 4 Stunden darauf wird ein wenig Suppe gegessen und darauf sich schlafen gelegt, da es dann erst am anderen Tage wirkt. Man muß aber die Vorsicht gebrauchen und nichts Rohes als: Salat, Milch oder dergleichen genießen.

Der tägliche Gebrauch ist 8–9 Tropfen für eine Mannesperson, 7 Tropfen für eine Frauensperson.

Alte Leute nehmen außer dem täglichen Gebrauch einen Löffel voll darüber.

#### Modern English version

Page 1

**Recipe from a Swedish doctor**

This recipe was found among the papers of a Swedish doctor by the name of Germes, who died at the age of 104 after falling from a horse. His family held this secret for several centuries, and these people lived exceptionally long lives as a result; his grandfather lived to be 130, his father 112, and his mother 107 years through the use of this elixir.

They took 7–8 drops of it every morning and evening with red wine, tea, or bouillon.

This recipe uses the following ingredients:

33 g = 1 *ounce* and a *Quentchen* of *Aloe hepatici*

4 g = 1 *Quentchen* of white turmeric

4 g = 1 *Quentchen* of saffron (of the best possible quality)

4 g = 1 *Quentchen* of gentian root

4 g = 1 *Quentchen* of agarikon

4 g = 1 *Quentchen* of rhubarb (of the best possible quality)

4 g = 1 *Quentchen* of Venetian theriac

45 g = 3 *Lots* of juniper electuary (paste-like)

All of these ingredients are pulverized and then passed through a fine-mesh sieve. Put the sieved powder, together with the theriac, into a large wine bottle, and then pour a measure of good brandy on top. Cover the wine bottle by tying a moist piece of parchment or a doubled bladder to the top, and once it has dried,

Page 2

Make a few holes with a needle so that the bottle does not burst open from the developing odors. Then leave the bottle in a dark place for 9 days, shaking it 2–3 times a day. On the 10th day, pour half of the liquid into a separate jar, taking care not to disturb the sediment at the bottom, and set the decanted liquid aside. Add another measure of brandy to the wine bottle and follow the same steps as before. On the 10th day, decant the liquid again and, if the original suspension starts to gain a cloudy appearance, filter it through blotting paper. Then mix all liquids, and store the elixir for use.

You can already begin using this elixir from the very first day.

Once you have started using it, you can expect to live long without having to undergo bloodletting or take other medicine. It strengthens the nerves, revives the spirits, sharpens the senses, stops the joints from shaking and the pain of a head cold, gets rid of heartburn, promotes good digestion, helps reduce bloating, quickly kills the worms and heals the wounds and abdominal colic, purifies the blood and promotes circulation of the same, is an infallible remedy for gout, promotes menstruation, restores lost coloring, and has a subtle and pain-free laxative effect if 2–3 doses are taken; in short, it is the restorer of all aspects of human health, even in the most decrepit of people, if it is used as specified below; it offers protection against contagious

Page 3

Diseases, it safely drives out the pox, and remarkably, it can be consumed in large doses without injurious effects. For the pox, it is a true *cordiali* if one gives the sufferer a full teaspoon in undiluted form for 9 days, each time with 7 spoonfuls of mutton soup. It also proved to be an effective remedy for dysentery.

The doses to be used for various symptoms are as follows:

1. Against heart palpitations 1 spoonful
2. indigestibility 2 spoonfuls in tea or coffee
3. Against intoxication 2 spoonfuls undiluted
4. For all symptoms of podagra 3 spoons undiluted
5. Against worms 1 full teaspoon daily for 8 days
6. Against dropsy 1 full teaspoon in red wine daily for one month
7. Against colic 2 spoonfuls in 4 spoonfuls of brandy
8. Against amenorrhea 1 spoonful in red wine daily for 3 days
9. Against fever of unknown origin 1 spoonful before the ague
10. Against dysentery 2 spoonfuls for a man-sized person, 1–3 spoonfuls in tea for small children
11. As a laxative the adult person should take 3 spoonfuls undiluted, then eat a small portion of soup 4 h later and go to bed, as it does not take effect until next day. However, one must take care not to consume anything raw, such as lettuce, milk or similar.

The daily dosage is 8–9 drops for a male person, 7 drops for female person.

Old people should take the daily dosage plus an additional spoonful.

### Analysis of the document from a historical–pharmacological perspective

The handwritten note contains a recipe for an herbal formulation. In the beginning of the document, the reader’s attention is caught by the story of Germes, a Swedish physician, whose past family members and himself are described as the intellectual property owners of a secret elixir for prolonged life that belonged to their family for several centuries. The writer of the note claims that the original recipe, finally revealing the secret of the potion to outsiders, was found in the jacket of Germes right after he fell off his horse and died at the age of 104. Due to the use of the preventive potion, the writer claims that Germes’ grandfather lived to be 130, his father 112, and his mother 107. Germes’ recipe precisely describes the ingredients of the potion, its preparation, and the required dosage for preventive use to prolong life. Additionally, the note provides precise explanations of how to administer the potion for immediate relief of various medical conditions such as colic, amenorrhea, fever, dysentery, constipation, intoxication, palpitations, flu-related pain, heartburn, wound care, blood cleansing, infectious diseases, and more.

#### Historical placement of Germes’ recipe

After transcription quality control regarding the exact spelling, completeness, and plausibility in the original document, the transcribed version of Germes’ recipe was further analyzed in regard to its historical placement. The estimated time of creation is between 1770 and 1820, based on the language and style of the German cursive handwriting. Nonetheless, this is merely a speculation as the beginnings of German cursive script, in this case specifically “Kurrentschrift,” can be dated back to the early fifteenth century [42, 43]. German cursive has been a style of handwriting that was commonly used in Germany and other German-speaking countries over the centuries [44, 45], known for its unique letterforms and connections between letters, giving it a distinct and elegant appearance. German cursive, known as "Kurrentschrift," was widely taught in schools until the 1940s when it was gradually replaced by Latin cursive [44, 46].

The authors estimate the historical placement of the primary source to be based on multiple indices, such as the spelling used in the document and the already practiced use of “Kurrent” script during that time. The calligraphy shows that writing was already commonplace for the scribe. Older documents from the 15th–17th centuries, which are still mostly written in the German chancery script (“Deutsche Kanzleischrift”) and are often characterized by a very arbitrary use of spelling and capitalization [36, 37], can be ruled out here. The spelling and terms used, as well as the use of certain techniques, also identify a specific time period which is further discussed in Sections 3.2.3, 3.2.4, and 3.2.5. Regarding the specialized terms of pharmacist language, it can be concluded that the European apothecary weights, such as the units "Quentchen” or “Lots," were in use from the middle of the thirteenth century until the beginning of the nineteenth century. These old units were gradually abolished starting from the end of the 18th century onward [47, 48].

#### Identification of units

Legible and complete written units in the handwritten recipe significantly facilitated comprehension of the measurements. Therefore, even though common in similar ancient documents of its time, it was not necessary to identify old alchemical symbols or units of measurement that are almost unknown today, e.g., with assistance of Kürner (1729) [17]. The following four units were used in the recipe: 1) ounce (“Unze”); 2) drachm (“Quentchen”); 3) lot (“Lot”); and 4) gram (“g”).

#### Contextualization regarding 18th- and 19th-century understandings of elixirs for longevity

Ancient medicine was deeply influenced by magical practices and beliefs [49]. During classical antiquity, magic charms, talismans, incantations, and rituals of magic and sorcery to avoid or cause sickness or to heal medical disorders co-existed with early medical concepts that abstained from these spiritual practices. For example, the Hippocratic medicine repudiated “unprofessional magic and wizardry” [50]. Thus, the sharp distinction between “overcoming outdated superstition” and advanced natural science in today’s time did already actually exist for roughly 2500 years. However, for the most part, neither the healers, practitioners, and mages, nor the patients distinguished between medicine and magic [51]. Elixirs and potions for longevity had significant impacts on ancient and medieval chemistry/alchemy and medicine in modern Germany, as noted by Weyer (2018) [52]. These mystic preparations were widely known as “Wundermittel” or “Wunderdrogen” (German for “panaceas,” “magic cures,” or “miracle drugs”) [53]. Another common term in late medieval and early modern Germany was “Wunderdrogentraktate” [54]. Traditional healers in local communities played a significant role in the contemporary health care system, possessing extensive knowledge of medicinal plants and the preparation of herbal remedies for various ailments. Herbal remedy knowledge, including recipes detailing harvesting times, preparation methods, and administration, was typically passed down orally from one generation to the next [55, 56]. The use of herbal medicine was deeply embedded in German culture and persisted alongside advances in modern medicine.

There was a significant increase in interest in potions and elixirs for longevity in the 17th and 18th centuries in Germany [57, 58]. These mystical formulations were usually created by individuals known as wise men or wise women, cunning folk, *Hexenmeister* (translated: “masters of witchcraft”) or *Kräuterhexen* (translated: "herb witches") [59]. Common ingredients involved psychedelic materials such as mandrake root or henbane, and common medicinal plants such as lavender, rosemary, or sage. Just as described in the note about Germes’ recipe, potions and elixirs for longevity were prepared using secret recipes that were passed down through generations. The popularity of potions and elixirs of longevity continued to grow in the 17th and 18th centuries due to the prevailing belief in magic and the desire to use the powers of such preparations for personal gain [50, 54, 60]. However, Germes’ recipe does not encompass beliefs in the supernatural or magic, such as claims that the elixir wards off evil spirits or even grants magical abilities. On the contrary, its claimed effect is of pharmacological nature, providing a remedy for medical disorders that were common to that particular time in history. It is possible that the terms "secret" and "inherited" were employed in the handwritten note strategically to enhance the perceived value of the manuscript, a practice that may have been employed several centuries ago to lend it greater significance and legitimacy.

Toward the end of the late Middle Ages, many of such elixirs and potions for longevity with medical claims were advertized by pharmacists via broadsheets. According to Schröder (2012) [54], precursors of such media can be traced back as far as the 12th century, i.e., still within the High Middle Ages, with evolving forms of public communication continuing throughout the Late Middle Ages and into the early modern period. In the following years, simplified replicas were produced by hand, indicating that Germes’ recipe originates from one such broadsheet. Often such recipes on herbal remedies have first been distributed in Latin writing (academic environments), then in the regional language, and finally orally in rural areas [61, 62]. In addition, there was an increasing emergence of handwritten commonplace books, providing basic recipes for all types of common medical disorders. In the beginning, these commonplace books were only available to aristocrats and rich townsmen. Upon upcoming availability of the letterpress, its distribution of such herbal books further increased [63]. Many of these printed books falsely attributed a long deceased, but famous, authority as the author, and collected content from various unknown sources unrelated to the claimed author. This phenomenon is called pseudepigraphy and has been reported for herbal books since antiquity (using the names of famous academics, such as Hippocrates, Dioscorides, and Galen, but also Apuleius, Macer, Albertus Magnus, Ortolf, and even still today with regard to Hildegard of Bingen) [64, 65]. Recipes such as Germes’ elixir remained popular during the 19th and early 20th centuries, despite changing fashions. Maria von Treben (1907–1991) was an Austrian herbalist known for popularizing traditional European herbal medicine through her widely read book *Health Through God’s Pharmacy*, which has influenced contemporary herbal practices despite limited scientific validation [66]. The questionable success of Maria von Treben in the late 20th century and until today provides proof that there is still plenty of interest in such recipes. Many of such folk and home remedies were initially derived from ancient academic medicine and previous old Latin writings [62, 67]. Germes’ elixir appears to be one of them.

In the handwritten note that describes Germes’ recipe, there are various terms that are no longer in use in the modern German language. Examples are: “Froste” (archaic term for cold fever/ague, see Grimm and Grimm (1866) [68]), “Blattern” (archaic term for smallpox, see [40]), “Zufällen” (translated into English as “coincidences;” however, this term was only used until the end of the 18th century and is correctly translated as “symptoms” or “everything that exceeds the normal conditions in a pathological way,” see Grimm and Grimm (1866) [68]), “Bouteille” (archaic term for wine bottle), “schüttet” (which would mean “pouring” in modern German, but intended meaning is “schüttelt,” which means “to shake”), “benimmt” (which would mean “to behave” in modern German, but intended meaning is “to take away” or “to stop”), “Verwahrungsmittel” (today, translated as “deposit,” but the contemporary meaning is “protection”), and “Mannsgroßen” (“Man-sized,” meaning an “adult”). Not only do terms change over time, but the spelling and grammar of a language changes also. Examples include: “Encian” (today “Enzian”), “Lattwerge” (today “Latwerge”), “Maaß” (today “Maß”), “darnach” (today “danach”), “läßt” (today “lässt”), “daß” (today “dass”), and “befundnes” (today “befundenes”). In Germes’ recipe, some sentences tend to be long in regard to today’s perception of German language, such as “Hierauf wird abermals ein Maaß Branntwein diesen Spezies zugegossen und wie das erste Mal verfahren, den 10. Tag hierauf abermals abgegossen, und wenn man merkt, daß es trübe werden will, so läßt man es durch Löschpapier laufen, man mischt dann beide Flaschen und bewahrt sie zum Gebrauch.”

The creator of the handwritten note seems to have been educated due to several indicators: (1) Illiteracy was still common; (2) they use Latin words, such as “secula” instead of “Jahrhunderte” (“centuries”) or “zurückgetretene Mensis” instead of “ausbleibende Menstruation” or “ausbleibende Menses”; and (3) they display medical knowledge, for example, by using the terms “Podagra” (foot gout) or “Cordiali” which is an archaic word for a cardiac agent that stimulates and strengthens one's heart, even though, other than in the 17th and 18th centuries, this would not be relatable to smallpox anymore according to modern understanding of the disease.

It is likely that Germes’ recipe has been inherited from a previous version of the remedy, which has then either been simplified, modified, and/or embellished with the stories of multiple family members who have lived to be over 100 years old. Hawthorn, which was utilized as a heart medication, had a similar story around 1900 (Petrovska, 2012). The actual story of the traditional medicinal uses of hawthorn is not widely known, as it has been distorted and elaborated upon through countless retellings, thus becoming a myth [69]. The document makes numerous claims about the effectiveness of Germes’ recipe, but it lacks evidence, as was customary during that period.

#### Analysis of potion ingredients

Table 1 presents the potion ingredients along with their biota, current accepted species, and family names.

**Table 1:** Ingredients reported in Germes’ recipe with their relevant species and family names

Potion ingredients	Biota	Scientific name	Family
*Aloe hepatici*	Plant	*Aloe vera* (L.) Burm.f	Xanthorrhoeaceae
White turmeric	Plant	*Curcuma zedoaria* (Christm.) Roscoe	Zingiberaceae
Saffron	Plant	*Crocus sativus* L	Iridaceae
Gentian root	Plant	*Gentiana lutea* L	Gentianaceae
Agarikon (larch tree sponge)	Tree fungus	*Fomitopsis officinalis* (Batsch) Bondartsev & Singer Until recently known as *Laricifomes officinalis* Vill.:Fr.) Kotl.et Pouzar	Fomitopsidaceae
Rhubarb	Plant	*Rheum rhabarbarum* L	Polygonaceae
Juniper electuary (a paste-like preparation produced from the berries)	Plant	*Juniperus communis* L	Cupressaceae
Venetian theriac	N/A	See Table 2 for ingredients	See Table 2 for family names
Brandy	N/A	N/A	N/A
Pig bladder (to cover and seal bottle during production)	N/A	N/A	N/A

Table 2 lists classical ingredients of the Venetian theriac.

**Table 2:** Classical ingredients of Venetian theriac [70, 71] and their relevant biota, accepted species, and family names

Venetian theriac classical ingredients	Biota	Scientific name	Family
Angelica root	Plant	*Angelica archangelica* L	Apiaceae
Snake knotweed	Plant	*Persicaria bistorta* (L.) Samp	Polygonaceae
Valerian	Plant	*Valeriana officinalis* L	Caprifoliaceae
Sea onion	Plant	*Drimia maritima* (L.) Stearn	Asparagaceae
Cinnamon	Plant	*Cinnamomum cassia* (L.) J. Presl	Lauraceae
Cardamom	Plant	*Elettaria cardamomum* (L.) Maton	Zingiberaceae
Myrrh	Plant	*Commiphora myrrha* (Nees) Engl	Burseraceae
White turmeric	Plant	*Curcuma zedoaria *(Christm.) Roscoe	Zingiberaceae
Iron(II) sulfate	N/A	N/A	N/A
Honey	N/A	N/A	N/A
Sherry (15% vol.)	N/A	N/A	N/A
Top with brandy	N/A	N/A	N/A

In its Germes’ formulation, there are seven medicinal ingredients: aloe, rhubarb, saffron, white turmeric, gentian, agarikon, and juniper. Juniper was added in a paste-like preparation (electuary) called “Latwerge” and *Aloe vera* as “Aloe hepatici” (see below). These were mixed in alcohol (brandy) together with some Venetian theriac, a composite medicine of classical origin. The herbal drugs used as ingredients of Germes’ recipe were common for Germany during the 17th and 18th centuries, and the Venetian theriac could be purchased in every reputable pharmacy during that time. These medicinal plants are also described as single ingredients for the treatment of diverse medical disorders in contemporary herbal books and before and after the 17th and 18th centuries [19–28].

Seemingly, one of the most important medicinal ingredients was Aloe hepatici (“Leber-Aloe,” translated: “liver aloe”). Aloe hepatici was produced via slow, gentle evaporation in the sun or in a vacuum, thereby creating a dull brown aloe hepatica type; rapid, strenuous evaporation would create the deep brown, glassy aloe lucida type with shiny broken surfaces. This process has already been described on pages 1226 and 1227 in Schröder et al. (1718) [34].

Venetian theriac was another major ingredient of Germes’ recipe that has also been previously combined with Aquae vitae, as seen on page 160 in Cordus (1598) [30], and its precursor *mithridatium*, respectively [72]. A slightly similar recipe, using theriac as the ingredient of “Aqua theriacalis,” was found on page 22 in Kürner (1729) [17]; however, there are no inclusions of Aloe and the other ingredients.

Other key ingredients seem to be rhubarb and larch sponge. There have been other recipes in the 17th century that include these two ingredients. For example, an “Extractum benedictum” has been described on page 261 in Schröder et al. (1685) [32]. According to the author, it has been used for purging “gallichte und schleimichte Feuchtigkeiten” (archaic term; in today’s understanding of German language “gallicht” would be translated as “bitter as bile,” “schleimichte” as “mucous,” and “Feuchtigkeiten” as “liquids”).

#### Analysis of the methods of preparation and administration

In Table 3, the methods of preparation and administration of Germes’ recipe are listed.

**Table 3:** Methods of preparation and administration of Germes’ recipe

Medical disorder	Amount prescribed	Dosage form
Heart palpitations	1 spoon	Pure
Indigestibility	2 spoons	In tea or coffee
Intoxication	2 spoons	Pure
Gout	3 spoons	Pure
Worms	1 teaspoon (daily) for 8 days	Pure
Dropsy	1 teaspoon (daily) for 1 month	In red wine
Colic	2 spoons	In 4 spoons of brandy
Delayed menstruation	1 spoon (daily) for 3 days	In red wine
Indeterminate fever	1 spoon	Before frost
Dysentery	Adults (“Man-sized persons”): 2 spoons Children: 1–3 spoons	Pure (adults) In tea (children)
In need of a laxative	Adults: 3 spoons	Pure, then eat a small portion of soup 4 h later and go to bed. The effect will be visible the next day (do not eat anything raw, such as milk or similar)
Smallpox	1 spoon daily for nine days	With 7 spoons of mutton soup
For general health and longer life (preventive use), as the potion “strengthens the nerves, revives the spirits, sharpens the senses, stops the joints from shaking and the pain of a head cold, gets rid of heartburn, promotes good digestion, helps reduce bloating, quickly kills the worms and heals the wounds and abdominal colic, purifies the blood and promotes circulation of the same, is an infallible remedy for gout, promotes menstruation, restores lost coloring and has a subtle and pain-free laxative effect if 2–3 doses are taken; in short, it is the restorer of all aspects of human health, even in the most decrepit of people, if it is used as specified [above]; it offers protection against contagious diseases, it safely drives out the pox, and remarkably, it can be consumed in large doses without injurious effects.”	Men: 8–9 drops daily Women: 7 drops daily Old people: daily dose as shown above, plus an additional spoon	Pure

The preparation of Germes’ elixir for prolonged life is characterized by a maceration procedure using ethyl alcohol in the form of brandy for extraction of bioactive secondary plant metabolites. This has been a common procedure, and similar formulations have been described in antiquity and the early Middle Ages. The so-called Lautertränke, herb macerations using wine, were prepared and administered depending on the season, sometimes even varying in its composition depending on the month. “Lautertränke” served for the preservation of health [73]. During the historical analysis of Germes’ recipe, one such preparation, “recipe 218” with an accurate description of its changing composition in each month of the year, was found in a handwritten herbal recipe book that was published as early as 511 CE [29, 74]. With improvements in distillation techniques came the invention of aqua vitae (“water of life” or brandy) by Taddeo Alderotti in the thirteenth century, which was further developed by Gabriel von Lebenstein in the 14th century, and Michael Puff and Hieronymus Brunschwig in the 15th century [75]. Following these developments, just as with Germes’ recipe, there were various botanical macerations using brandy as the extractant, whereas the herbal drug has often served as the name giver, such as Spirit of Melissa. The herbal books of Wirsung, (1568) [31] and Cordus (1598) [30] contain assortments of such botanical maceration recipes.

Germes’ recipe involves the use of animal bladders, such as pig bladders for sealing the maceration bottles during production period. In the 17th and 18th centuries, liquids stored in glass jars were often sealed with layers of pig bladder. During the 19th century, glass lids began replacing jar tops and were sealed with a tar-like substance, called pitch, or, more recently, synthetic materials like silicone rubber [76].

These are additional indicators when it comes to the historical placement of the handwritten note, most likely linking it to no later than the middle of the 19^th^ century.

During the literature review of old herbal books, none of the herbal remedies described distinguished between *Mannesperson* and *Frauensperson* (old word for “male person,” “female person,” respectively) in regard to the dosage administered. This observation appears to be quite uncommon or unusual.

### Assessment of uniqueness and origin of the primary source

At first glance, the handwritten note about Germes’ recipe seemed to be a unique piece of history. However, after careful review of the contemporary and earlier literature, multiple similarities in other recipes were discovered. For example, a slightly similar recipe, found on page 12 in Kürner (1729) [17], describes an “Aqua cordialis” or “Herzwässerchen” (translated: “Heart water”). On page 271 in Hellwig (1715) [33], an aqua vitae against colic is described, also possessing slightly similar ingredients.

Another handwritten document called, “Elixir de longue vie,” and classified as a unique piece in the Archives de la Ville et de l'Eurométropole de Strasbourg, depicts another very similar recipe with identical ingredients to that of Germes’ recipe [77]. This document has presumably been handwritten by the pharmacist Mr. Reeb from the Storcken Pharmacy in Strasbourg. Just like the elixir of love, this elixir of life, though more of a health potion, was famous and came with a steep price [77].

Toward the end of this study, a product still commercially available in Germany called “Schwedenbitter” (translated: “Swedish Bitters”) was discovered. The product description referenced an insert called, “The old manuscript,” from Maria Treben’s book, *Health Through God’s Pharmacy* [66]. Just like Germes’ recipe, it also interestingly describes an elixir for prolonged life that was found in the jacket of a Swedish doctor who fatally fell off his horse at the age of 104. In this recipe, the physician is not called Germes, but Dr. Samst. The legends say that the Swedish formula originates from Paracelsus, described as the genius recipe for Swedish Bitters, worldwide known as “elixir ad vitam longam” (translated: elixir for longevity). Its recipe is claimed to having been revived by Dr. Samst and his employee Dr. Hjärne in the 17th or 18th century. The Swedish Bitters being described lack herbs native to Sweden and rather use costly ingredients like saffron, zedoary, myrrh, aloe, and curcuma [78, 79]. These ingredients are nearly identical to those in Germes’ recipe. For example, the original Swedish Bitters formula does not include juniper electuary as in Germes’ recipe, but myrrh, yet myrrh is a common ingredient of Venetian theriac. Therefore, myrrh is likely also present and thus pharmacologically active in Germes’ recipe. Later, Maria Treben distinguished between The Great Swedish Bitters (original formula) and the Little Swedish Bitters, whereas the latter contained cheaper, more available ingredients. In her book, Maria Treben describes prescribing Swedish Bitters elixirs as sort of a panacea that actually works against a vast amount of medical disorders, some of which are also stated in Germes’ recipe [66]. Compared to the original preparations of the Swedish bitter, it is worth noting that the modern preparations of Swedish Bitters lack opium as an active ingredient of the Venetian theriac.

Schweizer (2003) [80] published a book about the medicinal plant *Aloe vera*. On page 75, the author informs about the story of an *Aloe vera*-based formula as an herbal remedy for prolonged life. In this case, the Swedish physician who fell off his horse and died is called Dr. Yernest. Just like in Germes’ recipe, Dr. Yernest died at the age of 104, his mother at 107, his father at 112, and his grandfather at 130. There is no information about the actual formula, other than Aloe being an ingredient, or on the methods of preparation and administration provided by the author. However, “Yernest” (Aloe formula) is phonetically more similar to “Germes” in comparison with “Samst” (Swedish Bitters).

In a recent study, Ahnfelt and Fors (2016) [81] meticulously investigated the origins of Swedish Bitters and claimed that its creation was during either the late 17th century or early 18th century (1720s–1740s) by Swedish physicians Urban Hiärne and Gustaf Lohrman. These two Swedish physicians sought permission from Charles XI, who was the King of Sweden at the time, to sell their remedies, but they never actually produced the medicines privately. In the first documentations, Swedish Bitters was called *Hiärnes Testamente* (Hiärne’s testament) [82]. Urban Hiärne is credited as the inventor of Swedish Bitters, although it remains unclear whether he truly invented it or whether it was actually created by someone who later became his family member. His and Lohrman’s papers list 26 remedies of which they claimed to have invented; however, none of these remedies included actual recipes. What was actually included were two descriptions of effects that indicated similar alignment to Swedish Bitters [81]. Generally, Hiärne kept secret the compositions of many of his inventions and only passed down secret information to his closest relatives. Urban Hiärne's two sons, Christian Henrik Hiärne and Ulric Leonhard Hiärne, traveled and sold medicine throughout Europe, particularly in Sweden and Germany, all the while promoting Swedish Bitters and making it fabulously famous. Oddly enough, this story aligns with that of Germes [82], and interestingly, the name "Hiärne" ("Hjärne" in German) is phonetically similar to "Germes." It remains unclear whether Urban, Christian Henrik, or Ulric Leonhard invented the original composition of Swedish Bitters, but it is quite likely that its creation came from one of them [81]. Regardless, it is important to note that roaming sellers of medicine and miracle cures play a large role in the admiration of Swedish Bitters, as the two brothers spread the word and the cure to those who otherwise might not have ever been fortunate enough to discover this brilliant elixir.

## Conclusion

In this study, a handwritten note containing Germes’ recipe for an elixir of life has been successfully transcribed from *Kurrentschrift* (a type of German cursive) to Latin alphabet-based German and then further translated into modern English. Based on various indicators such as the style of handwriting and medical terms and language style used, this primary source can be estimated to be written between 1770 and 1820. Unlike many elixirs for longevity during that period, it is worth mentioning that the note does not make use of magic or spiritualism in order to be effective. Its efficacy is claimed to be based on the medicinal properties of its ingredients and the accurate reproduction of its methods of preparation and administration for the treatment of various illnesses. The handwritten note and recipe seem to be unique to a certain degree. For example, the note states that the secret recipe originally belonged to a physician named “Germes,” which has not been found elsewhere according to the author’s best knowledge. However, during the 16th–18th centuries, pharmacists’ books and herbals did contain various similar recipes for longevity elixirs that incorporate nearly the same or very similar ingredients, along with ingredients that are used for the treatment of analog medical disorders to prolong life. What stands out is an herbal remedy that can still be purchased commercially today: The Swedish Bitters. Swedish Bitters likely has a history of 300 years as a medicine. Its originating story begins of a Swedish doctor that fatally fell of his horse at the age of 104, and his family members also died at very old age due to their use of the remedy often described as panacea. This tale is nearly identical to the one told in the handwritten note. However, the recipe of Swedish Bitters and other similar elixirs for prolonged life, such as Germes’ remedy, have changed over the course of time. Based on the history of Swedish Bitters described by Ahnfelt and Fors (2016), Burkl (2014), and Fetzner (2019) [78, 79, 81], it was possible to assess how the changing of historical contexts has affected the medicine’s composition over time. Consequently, Swedish Bitters are highly suitable for a biographical analysis of historical objects. The origins of it can be traced back to a well-documented era and location. It has been shown that Swedish Bitters rapidly gained popularity in the European market, particularly in Germany, where the handwritten note has been created, or rather where this elixir of life has been reproduced as Germes’ recipe, as well as further modified and embellished through retelling. During the early modern period, physicians and pharmacists often created and used their own medicines. The commercial sale of these secret compositions could be a lucrative business. Oftentimes, these physicians would modify existing formulas or recipes found in pharmacopeias and would conduct various experiments in their laboratories and on their patients. This practice allowed them to develop new medicines and refine their treatments [81]. Yet, as with most *Wundermittel* in history, its efficacy has often been assessed by its consumers through folklore and myths. Therefore, it is likely that, throughout Germany and Europe during the 17th and 18th centuries, Germes’ recipe acted as a home remedy for many, where it was originally being distributed by itinerant sellers of medicine and also by vendors of miracle cures, tricksters, charlatans, and quacks.

## Data Availability

No datasets were generated or analysed during the current study.
